# Endophytic Fungi Volatile Organic Compounds as Crucial Biocontrol Agents Used for Controlling Fruit and Vegetable Postharvest Diseases

**DOI:** 10.3390/jof10050332

**Published:** 2024-05-04

**Authors:** Lijun Ling, Lijun Feng, Yao Li, Rui Yue, Yuanyuan Wang, Yongpeng Zhou

**Affiliations:** 1 College of Life Science, Northwest Normal University, Lanzhou 730070, China; fenglj07101@163.com (L.F.); liyao9099@163.com (Y.L.); 17789613482@163.com (R.Y.); yywang0807@163.com (Y.W.); 2022212889@nwnu.edu.cn (Y.Z.); 2 Bioactive Products Engineering Research Center for Gansu Distinctive Plants, Northwest Normal University, Lanzhou 730070, China; 3 New Rural Development Research Institute, Northwest Normal University, Lanzhou 730070, China

**Keywords:** postharvest diseases, non-endophytic fungi, endophytic fungi, VOCs, biological control, control mechanisms, practical applications

## Abstract

Fruits and vegetables are an important part of the human diet, but during transportation and storage, microbial pathogens attack and spoil fruits and vegetables, causing huge economic losses to agriculture. Traditionally used chemical fungicides leave chemical residues, leading to environmental pollution and health risks. With the emphasis on food safety, biocontrol agents are attracting more and more attention due to their environmental friendliness. Endophytic fungi are present in plant tissues and do not cause host disease. The volatile organic compounds (VOCs) they produce are used to control postharvest diseases due to their significant antifungal activity, as well as their volatility, safety and environmental protection characteristics. This review provides the concept and characterization of endophytic fungal VOCs, concludes the types of endophytic fungi that release antifungal VOCs and their biological control mechanisms, as well as focuses on the practical applications and the challenges of applying VOCs as fumigants. Endophytic fungal VOCs can be used as emerging biocontrol resources to control postharvest diseases that affect fruits and vegetables.

## 1. Introduction

Fruits and vegetables are a crucial part of the human diet. They provide fiber, organic acids, vitamins, minerals, amino acids, and certain trace elements required by the body. Thus, they are rich in nutritional value and beneficial to human health [1,2]. However, it is important to note that fruits and vegetables can develop postharvest rot during various stages such as sorting, packaging, storage, transport, and marketing [3]. This is due to their susceptibility to mechanical damage, which can lead to the deposition of microbial pathogens on their surface. Under the right environmental conditions, these pathogens can directly invade fruits and vegetables through wounds, leading to postharvest rot [4]. Additionally, natural openings such as lenticels act as penetration sites [5]. Microbe-induced fruit and vegetable spoilage is a global problem. Fungal genera of *Alternaria*, *Aspergillus*, *Botrytis*, *Fusarium*, *Geotrichum*, *Gloeosporium*, *Monilinia*, *Penicillium*, *Mucor* and *Rhizopus* are the main causative pathogens of postharvest diseases of fruits and vegetables [6]. In addition to fruit and vegetable rot, these pathogenic fungi also produce other harmful metabolites, such as aflatoxin from *Aspergillus flavus* and ochratoxin A from *Aspergillus ochraceus*, some of which are also carcinogenic [7,8]. Thus, they pose huge safety risks to animals and humans [9]. According to the Food and Agriculture Organization of the United Nations (FAO), fruit and vegetable loss accounts for at least 40% of the total food loss produced annually worldwide [10]. Postharvest storage of fruits and vegetables is a major challenge.

Traditionally, chemical fungicides have been used as the main means of controlling or preventing the spoilage of postharvest fruits and vegetables. They include hydrogen peroxide, chlorine, trisodium phosphate, organic acids, and electrolytic water [11]. In addition, pesticides such as sterol biosynthesis inhibitors and imidazoline are registered for the control of postharvest diseases [12]. These chemical fungicides can kill microorganisms to some extent and prolong the shelf life of fresh produce. However, as people’s quality of life is improving, they are increasingly paying attention to the safety of agricultural products, which has gradually led to the withdrawal of chemical fungicides. Chlorine releases chlorine gas and produces carcinogenic by-products (trihalomethanes and haloacetic acids); hydrogen peroxide causes discoloration; and organic acids can damage human tissue and corrode equipment [13,14,15]. Chemical fungicides can also cause drug resistance in pathogenic microorganisms when used for a long period. For example, most citrus postharvest pathogens have developed substantial resistance to the commonly used fungicides thiamethoxam and imidazoline [16]. In addition, chemical fungicides can remain in the air, soil, plants, and other non-target organisms, affect water and soil quality, and cause considerable harm to plants, animals, and the environment [17]. Long-term direct or indirect pesticide exposure is a serious threat to human health, and the use of chemical fungicides needs to be reduced. Postharvest fungicide use is banned in some European countries or limited to a few registered chemicals [18].

Therefore, green environmental protection fungicides are becoming increasingly popular, and new safe fungicides are continually being sought to substitute chemical fungicides to reduce the occurrence of postharvest diseases in fruits and vegetables. In recent years, biological control has been a very promising direction for research and development. Biological control can effectively inhibit the attack of pathogens and has become a new trend in postharvest fruit and vegetable preservation [19,20]. As biological control agents, antagonistic microorganisms can use their structures and secondary metabolites to inhibit or kill pathogenic microorganisms, thereby significantly reducing the use of chemical fungicides [21]. Volatile organic compounds (VOCs) are released by microbes during their growth and reproduction and have been extensively studied for their apparent antifungal activity [22]. Ling et al. [23] found that VOCs from endophytic bacteria (such as *Bacillus* spp. and *Pseudomonas* spp.) have antifungal potential against numerous plant diseases. Compared with chemical fungicides, microbial VOCs have small molecular weights, are volatiles and quickly proliferate, and therefore do not easily remain on fruit and vegetable surfaces, which is beneficial for human health and environmental protection [24]. Second, VOCs are relatively safe, and their dosage is small enough to inhibit pathogenic fungi and are mostly harmless. 2,3-butanedione at 5 μL per plate effectively inhibited the mycelial growth, spore germination, and sporulation ability of wolfberry pathogenic fungi *Mucor circinelloides* LB1, *F*. *arcuatisporum* LB5, *Alternaria iridiaustralis* LB7, and *Colletotrichum fioriniae* LB8 [25]. Pathogen resistance is unlikely to emerge because microbial VOCs have a variety of resistance mechanisms against pathogenic fungi, such as via the inhibition of mycelial growth, spore germination, and the disruption of cell walls and cell membranes [26]. In addition, *T*. *asperellum* HbGT6-07 VOCs effectively reduced the colonial diameter, growth rate, and sclerotia production of two fungal pathogens: *Botrytis cinerea* (B05.10) and *Sclerotinia sclerotiorum* (A367) [27]. Consequently, microbial VOCs are promising biocontrol agents in terms of postharvest disease applications.

Endophytic fungi have received remarkable attention as microorganisms that can colonize the internal tissues of host plants without causing disease [28]. They colonize a wide range of plants and can be isolated from all plant organs, including roots, stems, leaves, flowers, fruits, and seeds [29]. Currently, some fungi with antagonistic effects, such as *Trichoderma* spp., are used in a large number of applications for controlling fruit and vegetable postharvest diseases, but the application regarding endophytes is very limited [30]. On the other hand, endophytic fungi are considered to have superior properties to non-endophytic fungi due to their better colonization ability and resistance to many biotic and abiotic stresses [31]. Endophytic fungi and their host plants have a mutually beneficial and symbiotic relationship. These fungi produce nutrients (polysaccharides, lipids, minerals and vitamins) and phytohormones, thereby promoting plant growth [32,33,34,35]. Second, they can improve plant resistance to stress, promote nutrient absorption by plants, and resist infection by pathogenic fungi [31]. Two endophytic fungi (*Penicillium citrinum* LWL4 and *Aspergillus terreus* LWL5) not only promote the growth of sunflower (*Helianthus annuus* L.) but also stimulate plant defense responses through the production of gibberellins, organic acids, and siderophore [36]. Endophytic fungal *Diaporthe* sp. CEL3 emits a characteristic scent (a fruity, sweet camphor odor) and inhibits the growth of 10 fungal pathogens from wide taxonomic groups like ascomycetes, basidiomycetes, and oomycetes [37]. Primary VOCs were generated by the endophytic fungus *Diaporthe apiculatum* strain FPYF 3052 and can suppress the growth of phytopathogenic fungi (*Alternaria alternata*, *Botryosphaeria dothidea*, *B*. *cinerea*, *Cercospora asparagi*, *Colletotrichum gloeosporioides*, *F*. *graminearum*, *Sphaeropsis sapinea*, and *Valsa sordida*), having inhibitory activities in the range of 23.8–66.7% within 24 h. Among the primary VOCs, commercial (−)-4-terpineol stood out as the terpenoid with the strongest inhibitory activity against these phytopathogenic fungi, with up to 100% inhibition [38]. Endophytic fungi can produce various VOCs with various biological activities, such as host plant growth promotion and antifungal, antioxidative, and antitumor activities, which are widely used in agriculture and industry [39]. Among them, VOCs released by endophytic fungi have strong fungicidal activity. This fungicidal activity is the main mechanism of antagonizing microbial pathogens after harvesting vegetables and has attracted significant research attention [40,41]. Naik [42] discusses the production of VOCs by endophytic fungi in fuel production and their potential applications in biological control. Studies conducted by Kaddes [41] have highlighted the importance of VOCs as antimicrobial agents. At present, research on the biocontrol mechanisms and practical applications of VOCs produced by endophytic fungi has not been sufficiently in-depth and has not been comprehensively reported yet.

In addition, Endophytic fungi release a wide variety of VOCs, which are still being explored in the study of mechanisms of resistance to pathogenic fungi. Therefore, special attention needs to be paid to the important role played by endophytic fungal VOCs in the control of fruit and vegetable postharvest diseases and to discuss the prospects and modalities of their application. Firstly, we here review the biological properties of endophytic fungal VOCs and their diversity, as well as types of endophytic fungi that release antifungal VOCs. Secondly, it also focuses on the mechanisms of endophytic fungal VOCs against pathogenic fungi, as well as practical applications of VOCs and future challenges and obstacles. Endophytic fungal VOCs play an important role in the postharvest disease control of fruits and vegetables as emerging biocontrol resources.

## 2. Endophytic Fungal Volatile Organic Compounds (VOCs)

Endophytic fungi colonize plant tissues and spend all or part of their life cycle in the host without inducing any noticeable symptoms of infection. They are widespread in nature and influence various biological activities in plants [43]. When endophytic fungi absorb various nutrients through catabolism and anabolism during the metabolic process, various fungal VOCs are finally produced [44]. VOCs are hydrophobic organic molecular compounds that evaporate into the gaseous phase at normal temperatures and pressures. They have a low molecular weight (<300 Da) and high vapor pressure (≥0.01 kPa at 20 °C). They are efficiently transported through air or soil when released [45]. VOCs can hence spread over a long distance at a high speed [45]. They are widely distributed in air, soil, water, animals, plants, etc., and can be used in ecosystems comprising various life forms such as microbes, plants, and insects. Thus, VOCs are crucial signaling molecules mediating the scientific niche [46,47].

Current studies on microbial VOCs usually use gas chromatography (GC) coupled with mass spectrometry (GC-MS) for VOC separation and identification [48]. For example, the use of SPME-GC/MS identified *D. apiculatum* strain FPYF 3052 as producing 15 volatile organic compounds (VOCs) mainly categorized as terpenes, benzene and benzene derivatives, alcohols, and hydrocarbons [38]. According to statistics, more than 250 different VOCs have been identified in fungi, mainly acids, alcohols, aldehydes, aromatics, esters, heterocycles, ketones, terpenes, and thiols [44]. These VOCs can be categorized into five types: terpenoids, fatty acid derivatives, benzene compounds, acetone, and amino acid derivatives. VOCs originate from different anabolic pathways, mainly the manganate/phenylalanine, mevalonate (MVA), methylerythritol phosphate (MEP), and lipoxygenase (LOX) pathways, which involve different enzymatic reactions to produce different VOC types [41]. According to incomplete statistics, more than 500 VOC-producing microbes have been identified and the VOCs of each strain vary with environmental variables, such as temperature [49]. In the future, more unique new VOCs will be discovered, and so, this emerging field of fungal endogenous VOCs is promising.

## 3. Endophytic Fungal Species Releasing VOCs of Antifungal Activity

In 2001, researchers isolated the endophytic fungus *Muscodor albus* from cinnamon trees, which produce five classes of VOCs (alcohols, esters, ketones, acids, and lipids) that are effective in inhibiting or killing multiple pathogenic microorganisms (*Aspergillus fumigatus*, *Candida albicans*, and *F*. *solani*, among others); esters are the most effective inhibitory compound class [50]. Researchers from various fields are keen on studying the fungal inhibitory activity of endophytic fungal VOCs. As summarized in Table 1, recent reports have indicated that endogenous fungal VOCs may be used as protectants against pathogens damaging stored fruits and vegetables. VOCs are safe and harmless to the human body within certain limits. For example, 2,3-butanedione, which is used as an edible additive, exhibited no hazards [51]. It was found that the concentration of volatile 6-pentyl-α-pyrone (6PP) of *T*. *atroviride* IC-11 was around 190 μg/mL, which had no adverse effect on human cells. Moreover, *T*. *atroviride* IC-11 VOCs were not cytotoxic to intestinal human colon carcinoma cells (Caco-2) [52]. Thus, owing to their advantages of rapid diffusion, safety, and effectiveness in biological control, endophytic fungal VOCs can be used as biological control agents.

**Figure 1:**
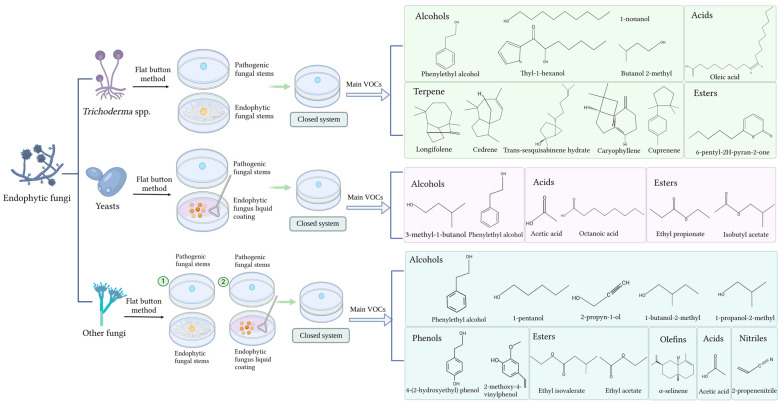
Chemical classification of endophytic fungal VOCs and their application methods. Numbers 1 and 2 represent the specific application of the other endophytic fungi flat button method in the first or second way. Created with BioRender.com.

The release of VOCs with antifungal activity produced by endophytic fungi is increasingly being reported. The main endophytic fungi that currently produce VOCs with antifungal activity are *Muscodor*, *Trichoderma* spp. and yeasts. The VOCs of *Muscodor* are a double-edged sword as they have been summarized as having major antifungal activity in Kaddes et al. [41] and Naik [42] as well as inhibitory effects on the growth of some plants (*Artemisia annua* seedlings) [72]. *Trichoderma* spp. are a particularly abundant source that can sustainably resist the growth of various phytopathogens [41,72]. Second, the release of antifungal active VOCs by yeasts, a generally abundant endophytic fungal species, has also received considerable research attention [55]. Therefore, this section focuses on *Trichoderma* spp. and yeasts. Therefore, Figure 1 summarizes the chemical classes of VOCs released by various endogenous fungi and their application methods.

### 3.1. Trichoderma spp.

*Trichoderma* spp. is a filamentous fungus exhibiting rapid mycelial growth and strong environmental adaptability. It serves as an antagonist of various phytopathogens and can be effectively used as a biocontrol agent [73,74,75]. *Trichoderma* spp. is among the most common fungi. It can settle asymptomatically in plant tissues, compete with harmful fungi for space and nutrition, improve the growth and defense functions of host plants [76], and play a crucial role in the prevention of pathogenic fungi by releasing antifungal active VOCs [41].

The endophytic fungus *T*. *koningiopsis* YIM PH30002 was isolated from the roots of a 2-year-old healthy sanqi (*Panax notoginseng*) plant. This fungus produced at least 10 VOCs, which were identified through the use of GC-MS as alkanes, monoterpenes, aromatic hydrocarbons, heterocycles, and aldehydes. These compounds could inhibit the root rot phytopathogenic fungi *Phoma herbar*, *F*. *flocciferum*, *Scytalidium lignicola*, and *Epicoccum nigrum* [67]. *T*. *koningiopsis* YIM PH30002 VOCs were expected to act as biological control agents for Panax ginseng root rot. Phenethyl alcohol, a VOC with antibacterial activity, could suppress postharvest rot caused by *F*. *incarnatum*. Phenylethyl alcohol produced by *T*. *asperellum* T76-14 can cause abnormal changes in the mycelium of the muskmelon pathogen *F*. *incarnatum*, leading to a 62.5% inhibition rate [77]. *T. spirale* T76-1 volatiles alcohol and pyran exhibited antifungal activity against *Corynespora cassiicola* and *Curvularia aeria*, with 41.29% and 42.35% inhibition rates [78]. Additionally, different strains of the same genus of the same plant differ in producing VOCs that exhibit different inhibitory effects. The isolation of seven T. virens strains from crop roots that release VOCs effectively inhibited the growth of the pathogenic fungus *Rhizoctonia solani*. However, VOCs from the seven strains exhibited different antifungal activities against *R. solani* strains. Among them, *T*. V3 and *T*. V4 displayed >50% inhibition at 5 days (52.8% and 59.4%), while inhibition caused by the remaining strains was below 50% [65].

### 3.2. Yeasts

Yeasts, unicellular fungi, rapidly colonize the surface of fruits and vegetables due to their high sugar content. They may produce extracellular polysaccharides to protect against pathogenic fungi and are resistant to long-term colonization under unfavorable conditions [79]. Yeasts interact with fruit and vegetable pathogens primarily through their antifungal activity, fungal parasitism, lytic enzyme production, induction of resistance, competition for scarce nutrients and space, and oxidative stress [80]. In addition, a growing body of research has found that endophytic yeasts can colonize plant tissues without harming the plant and can release various active VOCs that inhibit the growth and reproduction of fungal pathogens [55].

*Wickerhamomyces anomalus* (BS91), *Metschnikowia pulcherrima* (MPR3), *Aureobasidium pullulans* (PI1), and *Saccharomyces cerevisiae* (BCA61) strains release antifungal active VOCs, mainly ethanol and ethyl acetate. Furthermore, by incubating for 5 days at 25 °C, the yeast strains exhibited substantial oxygen consumption and carbon dioxide production. This acted synergistically with VOCs to exert antagonistic effects, thereby prolonging fruit and vegetable freshness [81]. The endophytic yeast *Geotrichum candidum* PF005, isolated from rice and wheat grains by Mitra et al. [58], releases the main VOC of ethyl isovalerate, which has significant antifungal activity and can reduce the formation of plant pathogens *Curvularia oryzae* and *Rhizoctonia solani* fungi nuclei, inhibit aerial mycelial development, and affect chitin distribution as well as mycelial and spore morphology. Alcohol acetyltransferases (AATs) catalyze ester formation between various alcohols and acetyl-CoA. The structure and function of AATs were investigated. They were found to be critical for the synthesis of antifungal volatile acetate by the endophytic fungi *G*. *candidum* PF005 [82]. The yeast *Hanseniaspora uvarum* 793 isolated from fig was used in in vivo experiments on strawberries and cherries because of its excellent biocontrol properties. VOCs released by *H*. *uvarum* 793 reduced the growth of *B*. *cinerea* at different temperatures (25 °C and 7 °C) [60]. The production of antifungal VOCs by yeast is a promising technology for extending the shelf life of fruits and reducing food waste and losses in the supply chain

## 4. Control Mechanism of Endophytic Fungal VOCs on Postharvest Fruit and Vegetable Diseases

Many microorganisms colonize fruit and vegetable surfaces before or after harvest. However, they generally cannot cause fruit and vegetable rot [83]. When fruits and vegetables are in an environment prone to invasion by pathogenic fungi, pathogenic fungi become extremely active, especially in the postharvest ripening period of fruits and vegetables [4]. Under suitable environmental conditions, these pathogenic microorganisms germinate, grow, multiply, and colonize rapidly, resulting in the postharvest rot of fruits and vegetables. Therefore, whether fungal VOCs can inhibit spore germination and hyphal growth is of great significance for fungal inhibition research [84]. Two strains of *Aureobasidium pullulans* (L1 and L8) produced VOCs that were efficient in preventing conidial growth of *P*. *expansum*, *P*. *digitatum*, and *P*. *italicum*. In vivo investigations revealed that 2-phenethyl alcohol, the primary component of L1 and L8 VOCs, could dramatically suppress lesions and reduce lesion diameter by >88% in *B*. *cinerea*-inoculated apples [53]. The endophytic fungi *T*. *asperellum* 6S-2 was isolated from apple tree roots, and 6-pentyl-2H-pyran-2-one (6-PP) was the main component of its volatile substance, accounting for 36.45% of its volatile substance. 6-PP could inhibit *Fusarium proliferatum* f. sp. *malus domestica* MR5, which causes apple replant disease, resulting in twisting, shrinking, swelling, and rupture [70]. VOCs of the endophytic fungi *Phaeosphaeria nodorum* could inhibit the mycelial growth of *Monilinia fructicola*, resulting in the narrowing of mycelial width [62].

Cell walls and membranes are crucial and essential tissue structures for microbes, with the function of protecting the cell and participating in the transport of substances. Cell wall and membrane integrity are critical for the survival of pathogenic fungi [85]. Endophytic fungal VOCs can damage the cell walls and membranes of pathogenic microorganisms, leading to changes in microbial morphology and leakage of contents and affecting microbial physiological function. A mixture of six endophytic fungal VOCs and alcohol synergistically alters the cell membrane permeability of the plant pathogen *F. oxysporum*. This causes a disruption in mycelial morphology as well as the inhibition of respiration and, eventually, the growth of the pathogen [86]. The VOCs of the endogenous fungus *Diaporthe* sp. CEL3 treat the pathogenic microorganism *Monilinia fructicola* and *Pythium ultimum*, causing the intracellular discharge of compounds of pathogenic microorganism, and with the extension of the processing time, the protein discharges also increase [37]. Some endophytic fungal VOCs can also kill pathogenic microorganisms by damaging their DNA. Single knockout testing revealed that the DNA repair, DNA metabolic activities, and stress response pathways of enzyme-deficient *Escherichia coli* are hypersensitive to *Muscodor albus* volatiles. VOCs prevent *Escherichia coli* from repairing damaged DNA, thereby preventing DNA replication or transcription. Second, VOCs can change *Escherichia coli* cell morphology, interfere with their selective permeability barrier, and make their cell membranes more permeable [87].

Endophytic fungal VOCs may act as signaling molecules that induce resistance in the plants that they colonize. They enhance the plant defense system to resist pathogens and promote plant growth. VOCs released by *T*. *asperloides* PSU-P1 increased the gene expression of the cell wall-degrading enzymes chitinase (CHI) and β-1,3-glucanase (GLU), as well as defense-related enzyme (peroxidase (POD)) activity in *Arabidopsis thaliana*, which is associated with increased oxidative stress in postharvest fruits and vegetables [88]. Similarly, 3-methyl-1-butanol, 1-decene, and 2-heptylfuran can improve the total chlorophyll content and fresh weight of *Arabidopsis thaliana* plants and promote their development [89]. The fungi *T*. *asperellum* T1 releases VOCs that increase the activity of cell wall-degrading enzymes, namely chitinase and β-1,3-glucanase, in lettuce. The pathogenic fungi cell wall undergoes morphological alterations owing to the accumulation of cell wall-degrading enzymes, thereby preventing the growth of the lettuce leaf spot-causing pathogens, *Corynespora cassiicola* and *Curvularia aeria*. Likewise, VOCs of *T*. *asperellum* T1 promote lettuce growth by increasing, for instance, the number of leaves and roots, plant biomass, and total chlorophyll content [71].

**Figure 2:**
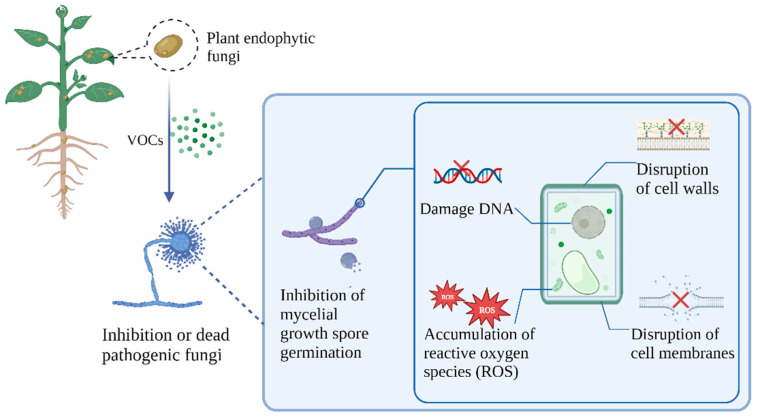
Mechanisms of the effects of endophytic fungal VOCs on postharvest diseases. Created with BioRender.com.

**Figure 3:**
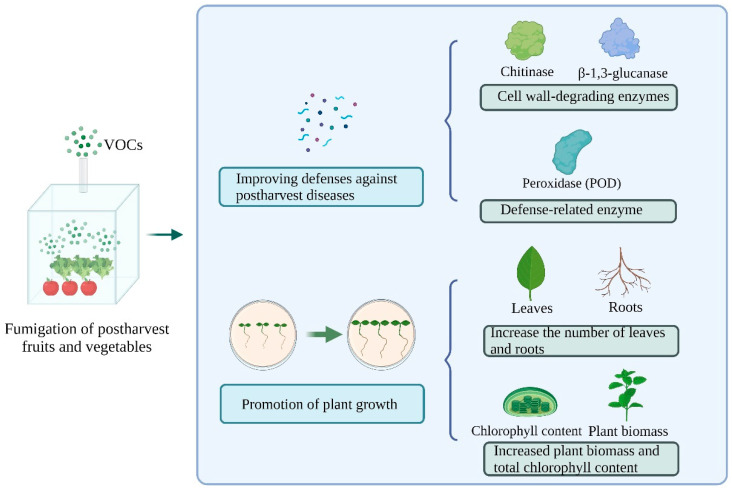
Endophytic fungal VOCs improve defense and promote the growth of fruits and vegetables. Created with BioRender.com.

An in-depth understanding of the specific physiological mechanism of the antifungal activity of endophytic fungal VOCs will help to comprehend the role and efficacy of fungal volatiles and their application in biocide research. Nowadays, the action mechanisms of fungal VOCs on pathogens of postharvest fruits and vegetables mainly include the inhibition of spore germination and mycelial growth of microbial pathogens (Figure 2); destruction of cell walls and membranes and change in the cell morphology of pathogens, which causes structural deformation, leakage of contents, and DNA damage and results in the impairment of physiological functions of pathogens (Figure 2); and the induction of resistance in fruits and vegetables by enhancing defense enzyme (peroxidase (POD)) activities, thus resisting postharvest diseases and promoting plant growth (Figure 3). Of course, fungal VOCs may act against pathogens through one or multiple mechanisms. *Sarocladium brachiariae* HND5 VOCs, for example, exhibit many fungi inhibitory mechanisms against detrimental microorganisms. It can destroy the cell wall and membrane of pathogenic fungi, leading to cell death. It can also trigger the production of plant chitinase and the accumulation of reactive oxygen species (ROS) in the pathogenic mycelium [63]. However, because of the diversity of fungi and their VOCs, the mechanisms of action are still poorly studied and need to be further explored. Some representative studies are summarized in Table 2.

## 5. Application of Endophytic Fungal VOCs as Postharvest Fruit and Vegetable Fumigants

As biological control mechanisms, endophytic fungal VOCs can be used to control the growth of plant diseases, thereby replacing chemical fungicides and addressing the issue of easy-lingering chemical fungicides in the environment and contaminating human health and the environment. Owing to their fast dispersion time, high efficiency, and other beneficial qualities, VOCs can be widely employed in agriculture. However, VOCs might be influenced by various circumstances during the application process. The production rate of VOCs is easily influenced by temperature, time, and culture substrate, as well as microbe-specific environmental conditions and even interactions with other species [86]. Whenever a fungus is cultivated in vitro, previously undiscovered VOCs are established, and VOCs vary with the age of the fungus and intraspecific and interspecific interactions [90]. The relative abundance of VOCs in *Xylaria* sp. PB3f3 varied with culture time and strain age. From the total VOCs, 25, 20, and 22 compounds were, respectively, produced at days 10, 20, and 30 of fungal growth [91]. Secondly, the different times and concentrations that VOCs access pathogen-infected fruits and vegetables lead to different inhibition effects. The endophytic fungus Aureobasidium *pullulans* L1, for example, exhibited good inhibitory activity against *Colletotrichum acutatum* and *P*. *expansum* for 12 h after apples were inoculated with the pathogenic fungus; introduced just 6 h after inoculation, it displayed the strongest inhibitory action against *B*. *cinerea* [92]. *Candida quercitrusa* Cq-1 VOCs effectively inhibited the growth of *Phytophthora infestans* mycelium, and the inhibition rate showed a linear relationship with the concentration of the fungi solution in the lower range. When the concentration of *C*. *quercitrusa* Cq-1 was 10^3^ CFU/mL, *Phytophthora infestans* mycelium barely grew, and the maximum inhibition was about 96.79% [54]. The optimal growth cycles of endophytic fungi are different, resulting in different concentrations of VOCs released during their growth and metabolism. Therefore, the use of optimum fumigation time and concentration will be effective in improving the inhibitory activity of endophytic fungal VOCs.

Fungi are diversified in terms of their generation of VOCs, comprising acids, alcohols, aldehydes, aromatics, esters, heterocycles, ketones, terpenes, thiols, and various other chemicals [44]. These compounds frequently mix to form complex combinations with inhibitory effects against one or more pathogenic fungi. VOCs from *Daldinia* cf. *concentrica*, for example, can effectively inhibit both mold growth on wheat seeds and *Aspergillus niger* infection in peanuts [56]. The volatile eucalyptol of the endophytic fungus *Nodulisporium* spp. CMU-UPE34 can control citrus fruit postharvest blight by inhibiting *P*. *digitatum* and *P*. *expansum* [93]. Microbial strains are susceptible to interference from various environmental factors. They are not susceptible to normal growth and metabolic activities, or even survival, under extreme acid–base conditions, too-high or too-low temperatures, extreme water scarcity, and UV radiation [94], which decreases the ability of microbes to produce VOCs and unstable effects. Consequently, additional studies need to be conducted on the biocontrol mechanism of VOCs, the screening of VOCs with broad-spectrum inhibitory activity and a high control effect, and environmentally resistant cultivars. Although VOCs do not easily stay on the fruit and vegetable surfaces, a limited percentage of fungal VOCs are detrimental. VOCs interfere with seed germination, seedling respiration, and root growth in *Amaranthus hypochondriacus*, *Panicum miliaceum*, *Trifolium pratense*, and *Medicago sativa* plants [95]. Therefore, when using endophytic fungal VOCs as control agents, it is necessary to consider whether VOCs have toxic effects on plants, damage plant cells, and affect their growth, metabolism, and physiological functions. It is necessary to find safe biological control agents.

On the market, products that control the consequences of vegetable harvesting are still controlled by physical methods, such as low-temperature storage, and chemical methods, such as using fludioxonil [96,97]. Concerning the use of endophytes, as a new concept, some preparations such as Candifruit^TM^, Shemer^TM^, and Boni-protect^TM^ have successfully prevented post-traumatic diseases [98]. Although products for endophytic fungal VOCs are not yet mature, research has been conducted on the subject of replacing chemical fungicides with endophytic fungal VOC fumigation to maintain the quality of postharvest fruits and vegetables. Because VOCs are easily volatilized in space, the problems of their airtightness and stability must be considered in the actual application process. Thus, in the laboratory, it is mainly taken as a flat plate to buckle and seal with a sealing film to prevent the volatilization of VOCs and avoid reducing their fungistatic activity [26]. In practical applications, depending on the type of fruits and vegetables and the storage conditions, fumigation can be used to achieve antagonistic effects against pathogenic fungi in a closed environment, such as entire storage rooms and individual transport containers [30]. In this case, endophytic fungi can be cultivated in a separate chamber, and the VOCs produced are released into the storage room via a pump without any direct contact with the fruits and vegetables, preventing infection of the fruits by the strain [40]. Instability caused by VOCs can be effectively reduced through closed environments. Secondly, VOCs produced by endophytic fungi can be incorporated into edible films and edible coatings and can also be used as ingredients in active packaging to effectively control microbial spoilage in fruits and vegetables and maintain fruit and vegetable quality [40]. Microencapsulation is a packaging technology that utilizes natural or synthetic polymer film-forming materials to encapsulate gases, liquids, or solids into particles with a particle size of 1–1000 μm [99]. Natural polymers, such as alginate, pectins, guar gums, and chitosan, are widely used as materials for the microencapsulation of many bioactive compounds [100]. Preparing *trans*-2-hexenal loaded polyurea microcapsules via an interfacial polymerization method effectively reduced the incidence of seed blight during wheat storage and prolonged the inhibition of pathogenic fungi [101]. By packaging VOCs in microcapsules, it is possible to effectively avoid the influence of environmental factors and to tackle the problem of VOC exposure and instability. In addition, sol-gel technology is used to encapsulate VOCs and can control the release rate of VOCs. Cross-linking during the sol-gel process can be controlled to produce a formulation with a constant release rate [102]. At present, VOC evaporation technology is still in the lab stage, and the development of economical, safe, and efficient technologies, such as microencapsulation and new packaging, will drive the industrialization and commercialization of VOCs.

In addition, the commercialization of endogenous fungal VOCs must take into account the originality of new technologies, as well as consistency and reliability under different production lines. Developing the product needs to relate to the business environment to solve its limitations and consider public acceptance. For large-scale production applications, the health assessment of the registration process needs to be fully clarified. Endophytic fungal VOCs still face great obstacles and challenges in terms of their future practical applications. (1) There is a wide variety of VOCs, some of which are even trace amounts, which are difficult to detect and characterize. (2) VOCs exert antifungal activity in the form of mixtures, and most of the current studies utilize single compounds in their pure form; therefore, it is necessary to study the antifungal activity of mixtures and explore whether there are synergistic or antagonistic effects between VOCs. (3) VOCs are highly unstable due to their volatility and can only be used in a closed environment. Therefore, the specific application model of VOC commercialization will become a complex issue and challenge in the future. (4) The mechanism of endophytic fungi VOCs to prevent and control postharvest diseases in fruits and vegetables is not comprehensive enough, and whether it affects the taste and nutrition of fruits and vegetables requires further research. (5) The safety of some VOCs is still a concern because workers who use endophytic fungi for fumigation with volatile organic compounds will inevitably inhale or be exposed to these volatile organic compounds during transportation and storage. (6) Most of the current antifungal applications of VOCs are still concentrated in the laboratory stage, and the gap between the application conditions in the laboratory and the factory is so large that it is a great challenge to realize the transition from the laboratory to the factory. The introduction of natural products into practice is complex, and aspects such as barriers to registration, difficulties in large-scale production, and industrial acceptance must be considered.

## 6. Conclusions

Endophytic fungi are isolated from plant tissues, and the use of the VOCs they produce to control postharvest diseases has received widespread attention. Currently, fungal species that release endophytic VOCs mainly include *Trichoderma* spp. and yeasts. Its mechanism of action is achieved via the disruption of the cellular and molecular structure of pathogenic fungi and enhancing the resistance of fruits and vegetables. The use of VOCs still faces many environmental factors and challenges in practical applications, and technical difficulties must be overcome to achieve commercialization. The application of endophytic fungi VOCs can effectively replace the harmful dependence on chemical fungicides and achieve guaranteed food safety in fruits and vegetables.

## Figures and Tables

**Table 1:** VOCs from endophytic fungi: main chemical components and effect against phytopathogenic fungi.

Endophytic Fungi	Endophytic Fungal Host Plants	Main VOCs	Pathogen	Pathogen Hosts	References
*Aureobasidium pullulans* (L1 and L8)	‘Redhaven’ peaches (*Prunus persica* (L.) Batsch)	2-phenethyl alcohol 1-butanol-3-methyl 1-butanol-2-methyl 1-propanol-2-methyl	*Botrytis cinerea* *Colletotrichum acutatum* *Penicillium expansum* *Penicillium digitatum* *Penicillium italicum*	‘Golden Delicious’ apples (*Malus domestica* L. Borkh) ‘Navel’ oranges (*Citrus sinensis* L. Osbeck)	[53]
*Candida* *quercitrusa strain* Cq-1	Litchi (*Litchi chinensis* Sonn.)	2-Phenylethanol	*Phytophthora infestans*	Potato (*Solanum tuberosum* L.)	[54]
*Candida nivariensis* DMKU-CE18	Leaves of rice (*Oryza sativa* L.), sugarcane (*Saccharum officenarum* L.) and corn (*Zea mays* L.)	1-pentanol	*Aspergillus flavus* A39	Corn grains (*Zea mays* L.)	[55]
*Daldinia* cf. *concentrica*	Olive tree (*Olea europaea* L.)	Alcohols Dienes Ketones Aldehydes Sesquiterpenes	*Molds* *Aspergillus niger*	Wheat grains (*Triticum aestivum* L.) Peanuts (*Arachis hypogaea* L.)	[56]
*Fusarium solani*-F4-1007	Argel (*Solenostemma arghel*)	3,4-dihydro-2H-1,5-(3″-t-butyl) benzodioxepine 4-(2-hydroxyethyl) phenol phenylethyl alcohol	*Cochliobolus spicifer*-CSN-20	Okra (*Abelmoschus esculentus*)	[57]
*Geotrichum candidum* PF005	Eggplant (*Solanum melongena*)	Ethyl isovalerate	*Rhizoctonia solani* *Curvularia oryzae*	Rice (*Oryza sativa* L.) Wheat (*Triticum aestivum* L.)	[58]
*Hypoxylon anthochroum* strains Blaeg1, Gseg1, Haeg2 and Smeg4	Burseraceae (*Bursera lancifolia*) Fabaceae (*Gliricidia sepium*) Celastraceae (*Hippocratea acapulcensis*) Euphorbiaceae (Sapium *macrocarpum*)	Sesquiterpenes Monoterpenes(eucalyptol)	*Fusarium oxysporum*	Cherry tomatoes (Solanum lycopersicum var. *cerasiforme*)	[59]
*Hanseniaspora uvarum* 793	Figs (*Ficus carica* L.)	Acids (acetic acid and octanoic acid) Esters (ethyl propionate, n-Propyl acetate, Isobutyl acetate, 2-methylbutyl acetate, furfuryl acetate, phenylmethyl acetate, 2-phenylethyl acetate)	*Botrytis cinerea*	Strawberries (*Fragaria × ananassa* Duch.) Cherries (*Prunus pseudocerasus* Lindl.)	[60]
*Nodulisporium* spp. CF016	Lauraceae trees (*Lauraceae* Juss.)	1-methyl-1,4-cyclo-hexadiene β-selinene α-selinene	*Botrytis cinerea* *Penicillium expansum*	Apple (*Malus pumila* Mill.)	[61]
*Phaeosphaeria nodorum*	Plum (*Prunus domestica*)	Ethyl acetate 3-methyl-1-butanol Acetic acid 2-propyn-1-ol 2-propenenitrile	*Monilinia fructicola*	Plum (*Prunus domestica*)	[62]
*Sarocladium brachiariae* HND5	The coastal grass	2-methoxy-4-vinylphenol 3,4-dimethoxystyrol Caryophyllene	*Fusarium oxysporum* f. sp. *cubense* (FOC)	Banana (*Musa nana* Lour.)	[63]
*Saccharomyces cerevisiae* NJ-1	Fig (*Ficus carica* L.)	3-methyl-1-butanol	*Aspergillus flavus*	Walnuts (*Juglans regia* L.)	[64]
*Trichoderma virens*	Crops	Sesquiterpenes (aromanderen, element, cadinene, and 2-Octanone) Monoterpene (limonene and bisnorhopane) Fatty acids (oleic acid, and monopalmtin) Caryophyllene Thojupsene	*Rhizoctonia solani*	Plants	[65]
*Trichoderma longibrachiatum* T (SP)-20	Groundnut (*Arachis hypogaea* L.)	Isolongifolan-7-ol Trans-sesquisabinene hydrate	*Sclerotium rolfsii*	Groundnut (*Arachis hypogaea* L.)	[66]
*Trichoderma koningiopsis* YIM PH30002	2-year-old healthy Sanqi (*Panax notoginseng*)	Alkanes Monoterpenes Aromatic hydrocarbons Heterocyclic Aldehydes	*Phoma herbarum* *Fusarium flocciferum* *Scytalidium lignicola* *Epicoccum nigrum*	Sanqi (*Panax notoginseng*)	[67]
*Trichoderma afroharzianum* strain MFLUCC19-0090 *Trichoderma afroharzianum* strain MFLUCC19-0091	*Schefflera leucantha* leaves	Phenylethyl alcohol	*Fusarium oxysporum* *Fusarium proliferatum*	Chili (*Capsicum annuum* L.)	[68]
*Trichoderma longibrachiatum* EF5	Rice (*Oryza sativa* L.)	Longifolene Caryophyllene Butanol 2-methyl Cedrene Cuprenene	*Sclerotium rolfsii* *Macrophomina phaseolina*	Plants	[69]
*Trichoderma asperellum* 6S-2	Roots of healthy apple (*Malus pumila* Mill.) trees	6-pentyl-2H-pyran-2-one	*Fusarium proliferatum* f. sp *malus domestica* MR5	Apple (*Malus pumila* Mill.)	[70]
*Trichoderma atroviride* IC-11	Rhizosphere of citrus(*Citrus reticulata* Blanco) tree	6-pentyl-α-pyrone	*Botrytis cinerea*	Blueberry (*Vaccinium* spp.)	[52]
*Trichoderma asperellum* T1	Lettuce (*Lactuca sativa*)	Thyl-1-hexanol 1-nonanol 6-pentyl-2H-pyran-2-one	*Corynespora cassiicola* *Curvularia aeria*	Lettuce (*Lactuca sativa*)	[71]

**Table 2:** The mechanisms of endophytic fungal VOCs in the control of postharvest diseases.

Main VOCs	Endophytic Fungi	Pathogen	Endophytic Fungal Usage and Dosage	Mechanisms	References
2-Phenylethanol	*Candida* *quercitrusa strain* Cq-1	*Phytophthora infestans*	20 µL cell concentration of 1 × 10^3^ CFU/mL	Inhibition of pathogen fungal mycelial development, blockage of the oxidative phosphorylation pathway	[54]
Ethyl isovalerate	*Geotrichum candidum* PF005	*Curvularia oryzae* *Rhizoctonia solani*	200 μL, OD600 = 5	Alteration of pathogen fungal mycelial morphology, influence on mycelial chitin distribution, and generation of oxidative stress	[58]
Ethyl acetate 3-methyl-1-butanol Acetic acid 2-propyn-1-ol 2-propenenitrile	*Phaeosphaeria nodorum*	*Monilinia fructicola*	5-mm-diameter plug of endophytic fungi	Reduced width of the pathogen fungal mycelial, causing disintegration of the mycelial content.	[62]
2-methoxy-4-vinylphenol 3,4-dimethoxystyrol Caryophyllene	*Sarocladium brachiariae* HND5	*Fusarium oxysporum* f. sp.	Plug of endophytic fungi	Induction of pathogenic fungal reactive oxygen species and chitinase gene accumulation and expression	[63]
3-methyl-1-butanol	*Saccharomyces cerevisiae* NJ-1	*Aspergillus flavus*	20 µL cell concentration of 1 × 10^7^ CFU/mL	Disruption of pathogen fungal cell membrane	[64]
6-pentyl-2H-pyran-2-one	*Trichoderma asperellum* 6S-2	*Fusarium proliferatum* f. sp *malus domestica* MR5	Plug of endophytic fungi	Destroys hyphae morphology and spore shape	[70]
Thyl-1-hexanol 1-nonanol 6-pentyl-2H-pyran-2-one	*Trichoderma asperellum* T1	*Corynespora cassiicola* *Curvularia aeria*	5-mm-diameter plug of endophytic fungi	Enhanced accumulation of cell wall degrading enzymes in lettuce	[71]
2-methyl-1-butanol 2-pentylfuran Acetic acid 6-pentyl-2H-pyran-2-one	*Trichoderma asperelloides* PSU-P1	*Colletotrichum* sp. *Corynespora cassiicola* *Curvularia lunata* *Ganoderma* sp. *Macrophomina phaseolina* *Neopestalotiopsis clavispora* *Penicillium oxalicum* *Sclerotium rolfsii* *Stagonosporosis cucurbitacearm*	5-mm-diameter plug of endophytic fungi	Antagonism of fungal pathogens, activation of plant defense responses, and promotion of plant growth	[88]
Longifolene Caryophyllene Butanol 2-methyl Cedrene Cuprenene	*Trichoderma longibrachiatum* EF5	*Sclerotium rolfsii*	8-mm-diameter plug of endophytic fungi	Alteration of mycelial structure	[69]
Sesquiterpenes (aromanderen, element, cadinene, and 2-Octanone) Monoterpene (limonene and bisnorhopane) Fatty acids (oleic acid, and monopalmtin) Caryophyllene Thojupsene	*Trichoderma virens*	*Rhizoctonia* *solani*	5-mm-diameter plug of endophytic fungi	Destruction of pathogenic fungal hyphae morphology	[65]

## Data Availability

Not applicable.

## References

[1] Jiang Q., Zhang M., Xu B. (2020). Application of Ultrasonic Technology in Postharvested Fruits and Vegetables Storage: A Review. Ultrason. Sonochem..

[2] Wallace T.C., Bailey R.L., Blumberg J.B., Burton-Freeman B., Chen C.O., Crowe-White K.M., Drewnowski A., Hooshmand S., Johnson E., Lewis R. (2020). Fruits, Vegetables, and Health: A Comprehensive Narrative, Umbrella Review of the Science and Recommendations for Enhanced Public Policy to Improve Intake. Crit. Rev. Food Sci. Nutr..

[3] Pétriacq P., López A., Luna E. (2018). Fruit Decay to Diseases: Can Induced Resistance and Priming Help?. Plants.

[4] Alegbeleye O., Odeyemi O.A., Strateva M., Stratev D. (2022). Microbial Spoilage of Vegetables, Fruits and Cereals. Appl. Food Res..

[5] Droby S., Zhimo V.Y., Wisniewski M., Freilich S. (2022). The Pathobiome Concept Applied to Postharvest Pathology and Its Implication on Biocontrol Strategies. Postharvest Biol. Technol..

[6] Dukare A.S., Paul S., Nambi V.E., Gupta R.K., Singh R., Sharma K., Vishwakarma R.K. (2019). Exploitation of Microbial Antagonists for the Control of Postharvest Diseases of Fruits: A Review. Crit. Rev. Food Sci. Nutr..

[7] Faria C.B., Santos F.C.D., Castro F.F.D., Sutil A.R., Sergio L.M., Silva M.V., Machinski Junior M., Barbosa-Tessmann I.P. (2017). Occurrence of Toxigenic Aspergillus Flavus in Commercial Bulgur Wheat. Food Sci. Technol..

[8] Zapaśnik A., Bryła M., Waśkiewicz A., Ksieniewicz-Woźniak E., Podolska G. (2022). Ochratoxin A and 2′R-Ochratoxin A in Selected Foodstuffs and Dietary Risk Assessment. Molecules.

[9] Perrone G., Ferrara M., Medina A., Pascale M., Magan N. (2020). Toxigenic Fungi and Mycotoxins in a Climate Change Scenario: Ecology, Genomics, Distribution, Prediction and Prevention of the Risk. Microorganisms.

[10] FAO (2011). Global Food Losses and Food Waste—Extent, Causes and Prevention.

[11] Chinchkar A.V., Singh A., Singh S.V., Acharya A.M., Kamble M.G. (2022). Potential Sanitizers and Disinfectants for Fresh Fruits and Vegetables: A Comprehensive Review. Food Process. Preserv..

[12] Ocampo-Suarez I.B., López Z., Calderón-Santoyo M., Ragazzo-Sánchez J.A., Knauth P. (2017). Are Biological Control Agents, Isolated from Tropical Fruits, Harmless to Potential Consumers?. Food Chem. Toxicol..

[13] Mani-López E., Palou E., López-Malo A. (2016). Effect of Different Sanitizers on the Microbial Load and Selected Quality Parameters of “Chile de Árbol” Pepper (*Capsicum frutescens* L.) Fruit. Postharvest Biol. Technol..

[14] De Corato U. (2020). Improving the Shelf-Life and Quality of Fresh and Minimally-Processed Fruits and Vegetables for a Modern Food Industry: A Comprehensive Critical Review from the Traditional Technologies into the Most Promising Advancements. Crit. Rev. Food Sci. Nutr..

[15] Khan I., Tango C.N., Miskeen S., Lee B.H., Oh D.-H. (2017). Hurdle Technology: A Novel Approach for Enhanced Food Quality and Safety—A Review. Food Control.

[16] Sánchez-Torres P., Tuset J.J. (2011). Molecular Insights into Fungicide Resistance in Sensitive and Resistant Penicillium Digitatum Strains Infecting Citrus. Postharvest Biol. Technol..

[17] Rani L., Thapa K., Kanojia N., Sharma N., Singh S., Grewal A.S., Srivastav A.L., Kaushal J. (2021). An Extensive Review on the Consequences of Chemical Pesticides on Human Health and Environment. J. Clean. Prod..

[18] Wisniewski M., Droby S., Norelli J., Liu J., Schena L. (2016). Alternative Management Technologies for Postharvest Disease Control: The Journey from Simplicity to Complexity. Postharvest Biol. Technol..

[19] Leneveu-Jenvrin C., Charles F., Barba F.J., Remize F. (2020). Role of Biological Control Agents and Physical Treatments in Maintaining the Quality of Fresh and Minimally-Processed Fruit and Vegetables. Crit. Rev. Food Sci. Nutr..

[20] Wang Z., Sui Y., Li J., Tian X., Wang Q. (2022). Biological Control of Postharvest Fungal Decays in Citrus: A Review. Crit. Rev. Food Sci. Nutr..

[21] Almeida O.A.C., De Araujo N.O., Dias B.H.S., De Sant’Anna Freitas C., Coerini L.F., Ryu C.-M., De Castro Oliveira J.V. (2023). The Power of the Smallest: The Inhibitory Activity of Microbial Volatile Organic Compounds against Phytopathogens. Front. Microbiol..

[22] Gong A.-D., Dong F.-Y., Hu M.-J., Kong X.-W., Wei F.-F., Gong S.-J., Zhang Y.-M., Zhang J.-B., Wu A.-B., Liao Y.-C. (2019). Antifungal Activity of Volatile Emitted from Enterobacter Asburiae Vt-7 against Aspergillus Flavus and Aflatoxins in Peanuts during Storage. Food Control.

[23] Ling L., Wang Y., Cheng W., Jiang K., Luo H., Pang M., Yue R. (2023). Research Progress of Volatile Organic Compounds Produced by Plant Endophytic Bacteria in Control of Postharvest Diseases of Fruits and Vegetables. World J. Microbiol. Biotechnol..

[24] Pereyra M.M., Garmendia G., Rossini C., Meinhardt F., Vero S., Dib J.R. (2022). Volatile Organic Compounds of Clavispora Lusitaniae AgL21 Restrain Citrus Postharvest Pathogens. Biol. Control.

[25] Ling L., Luo H., Zhao Y., Yang C., Cheng W., Pang M. (2023). Fungal Pathogens Causing Postharvest Fruit Rot of Wolfberry and Inhibitory Effect of 2,3-Butanedione. Front. Microbiol..

[26] Ling L., Pang M., Luo H., Cheng W., Jiang K., Wang Y. (2023). Antifungal Activity of Diacetyl, a Volatile Organic Compound, on Trichoderma Lixii F2 Isolated from Postharvest Lanzhou Lily Bulbs. Food Biosci..

[27] Kamaruzzaman M., Islam M.S., Mahmud S., Polash S.A., Sultana R., Hasan M.A., Wang C., Jiang C. (2021). In Vitro and in Silico Approach of Fungal Growth Inhibition by Trichoderma Asperellum HbGT6-07 Derived Volatile Organic Compounds. Arab. J. Chem..

[28] Newman D.J., Cragg G.M. (2015). Endophytic and Epiphytic Microbes as “sources” of Bioactive Agents. Front. Chem..

[29] Huang X., Ren J., Li P., Feng S., Dong P., Ren M. (2021). Potential of Microbial Endophytes to Enhance the Resistance to Postharvest Diseases of Fruit and Vegetables. J. Sci. Food Agric..

[30] Kumari M., Qureshi K.A., Jaremko M., White J., Singh S.K., Sharma V.K., Singh K.K., Santoyo G., Puopolo G., Kumar A. (2022). Deciphering the Role of Endophytic Microbiome in Postharvest Diseases Management of Fruits: Opportunity Areas in Commercial up-Scale Production. Front. Plant Sci..

[31] Samreen T., Naveed M., Nazir M.Z., Asghar H.N., Khan M.I., Zahir Z.A., Kanwal S., Jeevan B., Sharma D., Meena V.S. (2021). Seed Associated Bacterial and Fungal Endophytes: Diversity, Life Cycle, Transmission, and Application Potential. Appl. Soil Ecol..

[32] Chen Y., Mao W., Tao H., Zhu W., Qi X., Chen Y., Li H., Zhao C., Yang Y., Hou Y. (2011). Structural Characterization and Antioxidant Properties of an Exopolysaccharide Produced by the Mangrove Endophytic Fungus *Aspergillus* sp. Y16. Bioresour. Technol..

[33] Yang Y., Jin Z., Jin Q., Dong M. (2015). Isolation and Fatty Acid Analysis of Lipid-Producing Endophytic Fungi from Wild Chinese Torreya Grandis. Microbiology.

[34] Al-Obaidi J.R., Jambari N.N., Ahmad-Kamil E.I. (2021). Mycopharmaceuticals and Nutraceuticals: Promising Agents to Improve Human Well-Being and Life Quality. J. Fungi.

[35] Qin X., Xu J., An X., Yang J., Wang Y., Dou M., Wang M., Huang J., Fu Y. (2023). Insight of Endophytic Fungi Promoting the Growth and Development of Woody Plants. Crit. Rev. Biotechnol..

[36] Waqas M., Khan A.L., Hamayun M., Shahzad R., Kim Y.-H., Choi K.-S., Lee I.-J. (2015). Endophytic Infection Alleviates Biotic Stress in Sunflower through Regulation of Defence Hormones, Antioxidants and Functional Amino Acids. Eur. J. Plant Pathol..

[37] Santra H.K., Banerjee D. (2023). Antifungal Activity of Volatile and Non-Volatile Metabolites of Endophytes of Chloranthus Elatior Sw. Front. Plant Sci..

[38] Song X.-Y., Wang H., Ren F., Wang K., Dou G., Lv X., Yan D.-H., Strobel G. (2019). An Endophytic Diaporthe Apiculatum Produces Monoterpenes with Inhibitory Activity against Phytopathogenic Fungi. Antibiotics.

[39] Kanchiswamy C.N., Malnoy M., Maffei M.E. (2015). Chemical Diversity of Microbial Volatiles and Their Potential for Plant Growth and Productivity. Front. Plant Sci..

[40] Mari M., Bautista-Baños S., Sivakumar D. (2016). Decay Control in the Postharvest System: Role of Microbial and Plant Volatile Organic Compounds. Postharvest Biol. Technol..

[41] Kaddes A., Fauconnier M.-L., Sassi K., Nasraoui B., Jijakli M.-H. (2019). Endophytic Fungal Volatile Compounds as Solution for Sustainable Agriculture. Molecules.

[42] Naik B.S. (2018). Volatile Hydrocarbons from Endophytic Fungi and Their Efficacy in Fuel Production and Disease Control. Egypt. J. Biol. Pest Control.

[43] Kouipou Toghueo R.M., Boyom F.F. (2019). Endophytic Fungi from Terminalia Species: A Comprehensive Review. J. Fungi.

[44] Lemfack M.C., Gohlke B.-O., Toguem S.M.T., Preissner S., Piechulla B., Preissner R. (2018). mVOC 2.0: A Database of Microbial Volatiles. Nucleic Acids Res..

[45] Poveda J. (2021). Beneficial Effects of Microbial Volatile Organic Compounds (MVOCs) in Plants. Appl. Soil Ecol..

[46] Hung R., Lee S., Bennett J.W. (2015). Fungal Volatile Organic Compounds and Their Role in Ecosystems. Appl. Microbiol. Biotechnol..

[47] Effmert U., Kalderás J., Warnke R., Piechulla B. (2012). Volatile Mediated Interactions Between Bacteria and Fungi in the Soil. J. Chem. Ecol..

[48] Inamdar A.A., Morath S., Bennett J.W. (2020). Fungal Volatile Organic Compounds: More Than Just a Funky Smell?. Annu. Rev. Microbiol..

[49] Lemfack M.C., Nickel J., Dunkel M., Preissner R., Piechulla B. (2014). mVOC: A Database of Microbial Volatiles. Nucl. Acids Res..

[50] Sears J., Dirkse E., Markworth C., Strobel G.A. (2001). Volatile Antimicrobials from Muscodor Albus, a Novel Endophytic Fungus. Microbiology.

[51] Hallagan J.B. (2017). The Use of Diacetyl (2,3-Butanedione) and Related Flavoring Substances as Flavorings Added to Foods—Workplace Safety Issues. Toxicology.

[52] Bello F., Montironi I.D., Medina M.B., Munitz M.S., Ferreira F.V., Williman C., Vázquez D., Cariddi L.N., Musumeci M.A. (2022). Mycofumigation of Postharvest Blueberries with Volatile Compounds from Trichoderma Atroviride IC-11 Is a Promising Tool to Control Rots Caused by Botrytis Cinerea. Food Microbiol..

[53] Di Francesco A., Ugolini L., Lazzeri L., Mari M. (2015). Production of Volatile Organic Compounds by Aureobasidium Pullulans as a Potential Mechanism of Action against Postharvest Fruit Pathogens. Biol. Control.

[54] Lu J., Li J., Li L., Qi L., Wang Y., Yang S., Xu G., Dou D., Liu J., Wang X. (2023). Natural Product 2-Phenylethanol Inhibits ATP Synthesis of P. Infestans by Blocking the Oxidative Phosphorylation Pathway to Prevent Potato Late Blight. Postharvest Biol. Technol..

[55] Jaibangyang S., Nasanit R., Limtong S. (2020). Biological Control of Aflatoxin-Producing Aspergillus Flavus by Volatile Organic Compound-Producing Antagonistic Yeasts. BioControl.

[56] Liarzi O., Bar E., Lewinsohn E., Ezra D. (2016). Use of the Endophytic Fungus Daldinia Cf. Concentrica and Its Volatiles as Bio-Control Agents. PLoS ONE.

[57] Abdel-Motaal F.F., Kamel N.M., El-Sayed M.A., Abou-Ellail M. (2022). Biocontrol of Okra-Rot-Causing Cochliobolus Spicifer-CSN-20 Using Secondary Metabolites of Endophytic Fungi Associated with Solenostemma Arghel. Ann. Agric. Sci..

[58] Mitra M., Venkatesh P., Ghissing U., Biswas A., Mitra A., Mandal M., Mishra H.N., Maiti M.K. (2023). Fruity-Scented Antifungal Volatiles from Endophytic Geotrichum Candidum PF005: Broad-Spectrum Bioactivity against Stored Grain Pathogens, Mode of Action and Suitable Formulation for Mycofumigation. Biol. Control.

[59] Macías-Rubalcava M.L., Sánchez-Fernández R.E., Roque-Flores G., Lappe-Oliveras P., Medina-Romero Y.M. (2018). Volatile Organic Compounds from Hypoxylon Anthochroum Endophytic Strains as Postharvest Mycofumigation Alternative for Cherry Tomatoes. Food Microbiol..

[60] Ruiz-Moyano S., Hernández A., Galvan A.I., Córdoba M.G., Casquete R., Serradilla M.J., Martín A. (2020). Selection and Application of Antifungal VOCs-Producing Yeasts as Biocontrol Agents of Grey Mould in Fruits. Food Microbiol..

[61] Park M.-S., Ahn J.-Y., Choi G.-J., Choi Y.-H., Jang K.-S., Kim J.-C. (2010). Potential of the Volatile-Producing Fungus Nodulisporium Sp. CF016 for the Control of Postharvest Diseases of Apple. Plant Pathol. J..

[62] Pimenta R.S., Moreira da Silva J.F., Buyer J.S., Janisiewicz W.J. (2012). Endophytic Fungi from Plums (Prunus Domestica) and Their Antifungal Activity against Monilinia Fructicola. J. Food Prot..

[63] Yang Y., Chen Y., Cai J., Liu X., Huang G. (2021). Antifungal Activity of Volatile Compounds Generated by Endophytic Fungi Sarocladium Brachiariae HND5 against *Fusarium oxysporum* f. sp. *Cubense*. PLoS ONE.

[64] Yang T., Wang C., Li C., Sun R., Yang M. (2023). Antagonistic Effects of Volatile Organic Compounds of Saccharomyces Cerevisiae NJ-1 on the Growth and Toxicity of Aspergillus Flavus. Biol. Control.

[65] Inayati A., Sulistyowati L., Aini L.Q., Yusnawan E. Antifungal Activity of Volatile Organic Compounds from Trichoderma Virens. Proceedings of the International Conference on Biology and Applied Science (ICOBAS).

[66] Ayyandurai M., Akila R., Manonmani K., Harish S., Mini M.L., Vellaikumar S. (2023). Deciphering the Mechanism of Trichoderma Spp. Consortia Possessing Volatile Organic Compounds and Antifungal Metabolites in the Suppression of Sclerotium Rolfsii in Groundnut. Physiol. Mol. Plant Pathol..

[67] Chen J.-L., Liu K., Miao C.-P., Sun S.-Z., Chen Y.-W., Xu L.-H., Guan H.-L., Zhao L.-X. (2016). Salt Tolerance of Endophytic Trichoderma Koningiopsis YIM PH30002 and Its Volatile Organic Compounds (VOCs) Allelopathic Activity against Phytopathogens Associated with *Panax notoginseng*. Ann. Microbiol..

[68] Khruengsai S., Pripdeevech P., D’Souza P.E., Panuwet P. (2021). Biofumigation Activities of Volatile Compounds from Two *Trichoderma Afroharzianum* Strains against *Fusarium* Infections in Fresh Chilies. J. Sci. Food Agric..

[69] Sridharan A.P., Thankappan S., Karthikeyan G., Uthandi S. (2020). Comprehensive Profiling of the VOCs of Trichoderma Longibrachiatum EF5 While Interacting with Sclerotium Rolfsii and Macrophomina Phaseolina. Microbiol. Res..

[70] Wang H., Zhang R., Duan Y., Jiang W., Chen X., Shen X., Yin C., Mao Z. (2021). The Endophytic Strain Trichoderma Asperellum 6S-2: An Efficient Biocontrol Agent against Apple Replant Disease in China and a Potential Plant-Growth-Promoting Fungus. J. Fungi.

[71] Wonglom P., Ito S., Sunpapao A. (2020). Volatile Organic Compounds Emitted from Endophytic Fungus Trichoderma Asperellum T1 Mediate Antifungal Activity, Defense Response and Promote Plant Growth in Lettuce (*Lactuca sativa*). Fungal Ecol..

[72] Zhi-Lin Y., Yi-Cun C., Bai-Ge X., Chu-Long Z. (2012). Current Perspectives on the Volatile-Producing Fungal Endophytes. Crit. Rev. Biotechnol..

[73] Tchameni S.N., Cotârleț M., Ghinea I.O., Bedine M.A.B., Sameza M.L., Borda D., Bahrim G., Dinică R.M. (2020). Involvement of Lytic Enzymes and Secondary Metabolites Produced by *Trichoderma* spp. in the Biological Control of *Pythium myriotylum*. Int. Microbiol..

[74] Siebatcheu E.C., Wetadieu D., Youassi Youassi O., Bedine Boat M.A., Bedane K.G., Tchameni N.S., Sameza M.L. (2023). Secondary Metabolites from an Endophytic Fungus *Trichoderma Erinaceum* with Antimicrobial Activity towards *Pythium ultimum*. Nat. Prod. Res..

[75] Shafiq M., Bakht J., Iqbal A., Shafi M. (2020). Growth, Protein Expression and Heavy Metal Uptake by Tobacco under Heavy Metals Contaminated Soil. Pak. J. Bot..

[76] Stracquadanio C., Quiles J.M., Meca G., Cacciola S.O. (2020). Antifungal Activity of Bioactive Metabolites Produced by Trichoderma Asperellum and Trichoderma Atroviride in Liquid Medium. J. Fungi.

[77] Intana W., Kheawleng S., Sunpapao A. (2021). Trichoderma Asperellum T76-14 Released Volatile Organic Compounds against Postharvest Fruit Rot in Muskmelons (Cucumis Melo) Caused by *Fusarium incarnatum*. J. Fungi.

[78] Baiyee B., Pornsuriya C., Ito S., Sunpapao A. (2019). Trichoderma Spirale T76-1 Displays Biocontrol Activity against Leaf Spot on Lettuce (*Lactuca sativa* L.) Caused by *Corynespora cassiicola* or *Curvularia aeria*. Biol. Control.

[79] Madbouly A.K., Abo Elyousr K.A.M., Ismail I.M. (2020). Biocontrol of Monilinia Fructigena, Causal Agent of Brown Rot of Apple Fruit, by Using Endophytic Yeasts. Biol. Control.

[80] Spadaro D., Droby S. (2016). Development of Biocontrol Products for Postharvest Diseases of Fruit: The Importance of Elucidating the Mechanisms of Action of Yeast Antagonists. Trends Food Sci. Technol..

[81] Contarino R., Brighina S., Fallico B., Cirvilleri G., Parafati L., Restuccia C. (2019). Volatile Organic Compounds (VOCs) Produced by Biocontrol Yeasts. Food Microbiol..

[82] Mitra M., Singh R., Ghissing U., Das A.K., Mitra A., Maiti M.K. (2022). Characterization of an Alcohol Acetyltransferase GcAAT Responsible for the Production of Antifungal Volatile Esters in Endophytic Geotrichum Candidum PF005. Microbiol. Res..

[83] Ssemanda J.N., Reij M.W., van Middendorp G., Bouw E., van der Plaats R., Franz E., Muvunyi C.M., Bagabe M.C., Zwietering M.H., Joosten H. (2018). Foodborne Pathogens and Their Risk Exposure Factors Associated with Farm Vegetables in Rwanda. Food Control.

[84] Schreuder W., du Plooy W., Erasmus A., Savage C., Basson E., Lennox C., Fourie P.H. (2018). Postharvest Fungicide Treatments and Cold Storage Control Citrus Black Spot Infections. Crop Prot..

[85] Zhao X., Zhou J., Tian R., Liu Y. (2022). Microbial Volatile Organic Compounds: Antifungal Mechanisms, Applications, and Challenges. Front. Microbiol..

[86] Medina-Romero Y.M., Roque-Flores G., Macías-Rubalcava M.L. (2017). Volatile Organic Compounds from Endophytic Fungi as Innovative Postharvest Control of Fusarium Oxysporum in Cherry Tomato Fruits. Appl. Microbiol. Biotechnol..

[87] Alpha C.J., Campos M., Jacobs-Wagner C., Strobel S.A. (2015). Mycofumigation by the Volatile Organic Compound-Producing Fungus Muscodor Albus Induces Bacterial Cell Death through DNA Damage. Appl. Environ. Microbiol..

[88] Phoka N., Suwannarach N., Lumyong S., Ito S., Matsui K., Arikit S., Sunpapao A. (2020). Role of Volatiles from the Endophytic Fungus Trichoderma Asperelloides PSU-P1 in Biocontrol Potential and in Promoting the Plant Growth of *Arabidopsis thaliana*. J. Fungi.

[89] Lee S., Behringer G., Hung R., Bennett J. (2019). Effects of Fungal Volatile Organic Compounds on *Arabidopsis thaliana* Growth and Gene Expression. Fungal Ecol..

[90] Sánchez-Fernández R.E., Diaz D., Duarte G., Lappe-Oliveras P., Sánchez S., Macías-Rubalcava M.L. (2016). Antifungal Volatile Organic Compounds from the Endophyte *Nodulisporium* sp. Strain GS4d2II1a: A Qualitative Change in the Intraspecific and Interspecific Interactions with *Pythium aphanidermatum*. Microb. Ecol..

[91] Sánchez-Ortiz B.L., Sánchez-Fernández R.E., Duarte G., Lappe-Oliveras P., Macías-Rubalcava M.L. (2016). Antifungal, Anti-Oomycete and Phytotoxic Effects of Volatile Organic Compounds from the Endophytic Fungus *Xylaria* sp. Strain PB3f3 Isolated from *Haematoxylon Brasiletto*. J. Appl. Microbiol..

[92] Mari M., Martini C., Spadoni A., Rouissi W., Bertolini P. (2012). Biocontrol of Apple Postharvest Decay by Aureobasidium Pullulans. Postharvest Biol. Technol..

[93] Suwannarach N., Kumla J., Bussaban B., Nuangmek W., Matsui K., Lumyong S. (2013). Biofumigation with the Endophytic Fungus Nodulisporium Spp. CMU-UPE34 to Control Postharvest Decay of Citrus Fruit. Crop Prot..

[94] Stolz J.F. (2017). Gaia and Her Microbiome. FEMS Microbiol. Ecol..

[95] Ulloa-Benítez Á., Medina-Romero Y.M., Sánchez-Fernández R.E., Lappe-Oliveras P., Roque-Flores G., Duarte Lisci G., Herrera Suárez T., Macías-Rubalcava M.L. (2016). Phytotoxic and Antimicrobial Activity of Volatile and Semi-Volatile Organic Compounds from the Endophyte *Hypoxylon Anthochroum* Strain Blaci Isolated from *Bursera Lancifolia* (Burseraceae). J. Appl. Microbiol..

[96] Brandhorst T.T., Klein B.S. (2019). Uncertainty Surrounding the Mechanism and Safety of the Post-Harvest Fungicide Fludioxonil. Food Chem. Toxicol..

[97] Fallik E., Ilić Z. (2021). The Influence of Physical Treatments on Phytochemical Changes in Fresh Produce after Storage and Marketing. Agronomy.

[98] Fenta L., Mekonnen H., Gashaw T. (2020). Biocontrol Potential of Trichoderma and Yeast against Post Harvest Fruit Fungal Diseases: A Review. Int. J. Life Sci..

[99] Rathore S., Desai P.M., Liew C.V., Chan L.W., Heng P.W.S. (2013). Microencapsulation of Microbial Cells. J. Food Eng..

[100] Chen L., Gnanaraj C., Arulselvan P., El-Seedi H., Teng H. (2019). A Review on Advanced Microencapsulation Technology to Enhance Bioavailability of Phenolic Compounds: Based on Its Activity in the Treatment of Type 2 Diabetes. Trends Food Sci. Technol..

[101] Ma D., Zhang T., Wang G., Cao C., Mu W., Li B., Dou D., Liu F. (2023). Polyurea Microcapsule Encapsulation Improves the Contact Toxicity, Inhibition Time and Control Effect of Trans-2-Hexenal against Fusarium Graminearum. Ind. Crops Prod..

[102] Sharifi R., Ryu C.-M. (2018). Biogenic Volatile Compounds for Plant Disease Diagnosis and Health Improvement. Plant Pathol. J..

